# Immunogenic selection of HIV-1 MPER epitopes for improved vaccine design

**DOI:** 10.1186/1742-4690-9-S2-P322

**Published:** 2012-09-13

**Authors:** L Wieczorek, M Rao, K Peachman, N Steers, M Marovich, N Michael, V Polonis, V Rao

**Affiliations:** 1 Henry M. Jackson Foundation, Silver Spring, MD, USA; 2 Walter Reed Army Institute of Research, Silver Spring, MD, USA; 3 Catholic University of America, Washington, DC, USA

## Background

The goal of this study was to design structural mimics of HIV-1 epitopes that have the potential to induce broadly neutralizing antibodies (bnAbs). The structure of the gp41 membrane proximal external region (MPER), targeted by three bnAbs, requires further definition. Experiments were designed to select epitopes with enhanced binding to MPER bnAbs, to identify neutralization-competent MPER structures, and to determine if selected MPER epitopes can broaden the immune response as potential vaccines.

## Methods

MPER epitopes were selected by biopanning with phage-displayed peptide libraries against bnAbs 4E10, 2F5 and Z13. Epitopes were screened in competition binding assays where M13-displayed epitopes competed with envelope peptides or infectious HIV-1 for antibody binding. In vivo response to MPER was assessed by M13 immunoprecipitation and neutralization competition assays using HIV-positive plasma, and immunogenicity of select epitopes was assessed in BALB/c mice.

## Results

Forty-six unique 4E10 epitopes were identified, representing both MPER homologous and non-homologous sequences. Fourteen epitopes, capable of competing with MPER peptide and HIV-1 for 4E10 binding, bound HIV-positive IgG. Of these, five epitopes absorbed MPER-specific neutralizing activity in HIV-positive patient plasma. Mouse immunization with the selected neutralization-competent MPER epitopes elicited HIV-1 specific cellular and humoral immune responses and boosted the neutralizing activity of a gp145 Env subunit vaccine.

## Conclusion

This study demonstrates the diversity of epitope recognition by 4E10 and the unique response to MPER in vivo. The M13-displayed, neutralization-competent structures of the 4E10 epitopes have the potential to elicit and boost HIV-1 neutralizing antibody production in mice. Phage-displayed epitopes can rapidly and inexpensively be selected to provide epitope-specific depth and variation to HIV-1 vaccine designs without requiring modification to major vaccine components.

